# Primary breast diffuse large B-cell lymphoma in a patient with systemic lupus erythematosus: A case report and review of the literature

**DOI:** 10.1097/MD.0000000000021736

**Published:** 2020-08-14

**Authors:** Feifei Shen, Gang Li, Huifeng Jiang, Shupeng Zhao, Fengjie Qi

**Affiliations:** a Department of Pathology; b Department of Orthopedics, Qilu Hospital (Qingdao), Cheeloo College of Medicine, Shandong University, Qingdao, Shandong; c Department of Thyroid and Breast Surgery; d Department of Pathology, Luohu District People's Hospital, Shenzhen, China.

**Keywords:** breast, diffuse large B-cell lymphoma, immunosuppressive drugs, SLE, systemic lupus erythematosus

## Abstract

**Rationale:**

Pilot studies have reported that patients with systemic lupus erythematosus (SLE) appear more likely to develop into neoplasia, especially lymphatic hyperplasia diseases. To our knowledge, this is the first case report of the concomitant onset of SLE and primary breast diffuse large B-cell lymphoma (PB-DLBCL).

**Patient concerns:**

We reported an unusual case of the occurrence of primary breast diffuse large B-cell lymphoma in a 25-year-old female patient who had been diagnosed with SLE and treated with immunosuppressive drugs for about 4 years. She presented a 7-week history of a painless mass above the left breast and no history suggestive of any nipple discharge, fever, and weight loss.

**Diagnosis:**

Ultrasonography of the breast showed that there was 1 mass in the left breast. After breast mass surgical resection, histopathological examinations were performed and revealed that it was primary breast diffuse large B-cell lymphoma.

**Interventions:**

Treatment strategy with vincristine and dexamethasone was used to improve symptoms. However, the patient's renal function deteriorated and the blood potassium rose continuously and she and their family members refused the follow-up treatments.

**Outcomes:**

The patient died 8 months after she was discharged from the hospital.

**Lessons:**

PB-DLBCL is a rare occurrence in SLE patients. Therefore, a careful examination is very important in SLE cohort, as activity of the disease and malignancy may mimic each other. Meanwhile, when symptoms cannot be explained or insensitive to treatment, the occurrence of malignant tumors must be highly considered.

## 1 Introduction

Systemic lupus erythematosus (SLE) is an autoimmune inflammatory connective tissue disease involving multiorgans that predominantly affects the women in the child-bearing period.^[1,2]^ Numerous case reports and epidemiologic studies indicated that there was a certain relationship between SLE and non-Hodgkin lymphoma, primarily of the diffuse large B cell type (DLBCL).^[2–8]^ Nevertheless, to our knowledge, this is the first case report of the concomitant onset of SLE and primary breast diffuse large B-cell lymphoma (PB-DLBCL). Here, we described an SLE patient with PB-DLBCL and review the literature.

## 2 Case report

**Figure 1:**
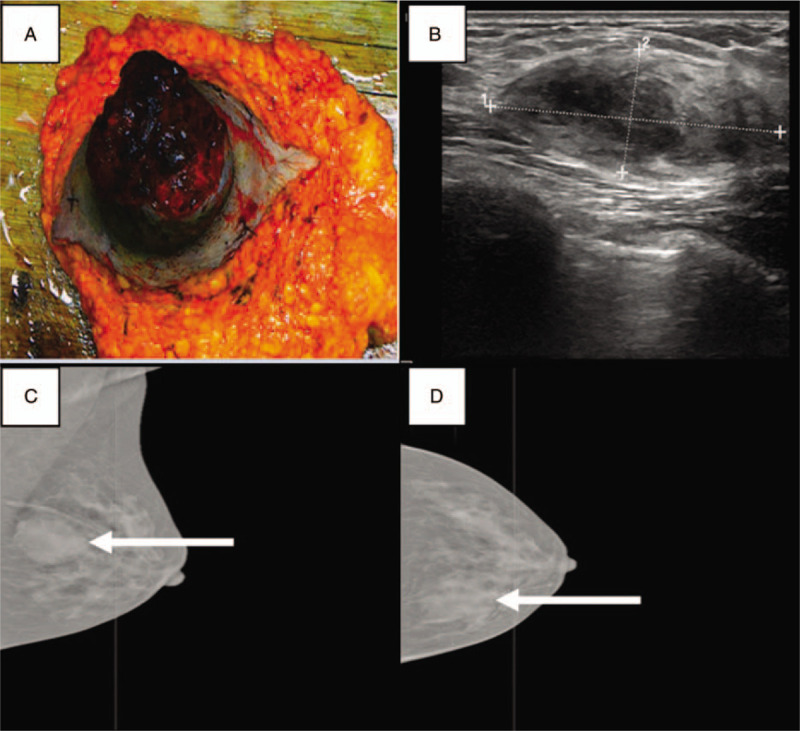
A, Diffuse large B-cell lymphoma in the left breast with multiple necrotic and hemorrhagic foci. B, The ultrasonography result showed that low echo nodule in the left breast. C and D, Molybdenum palladium roentgenograph of left breast revealed an irregular soft tissue mass (arrow).

**Figure 2:**
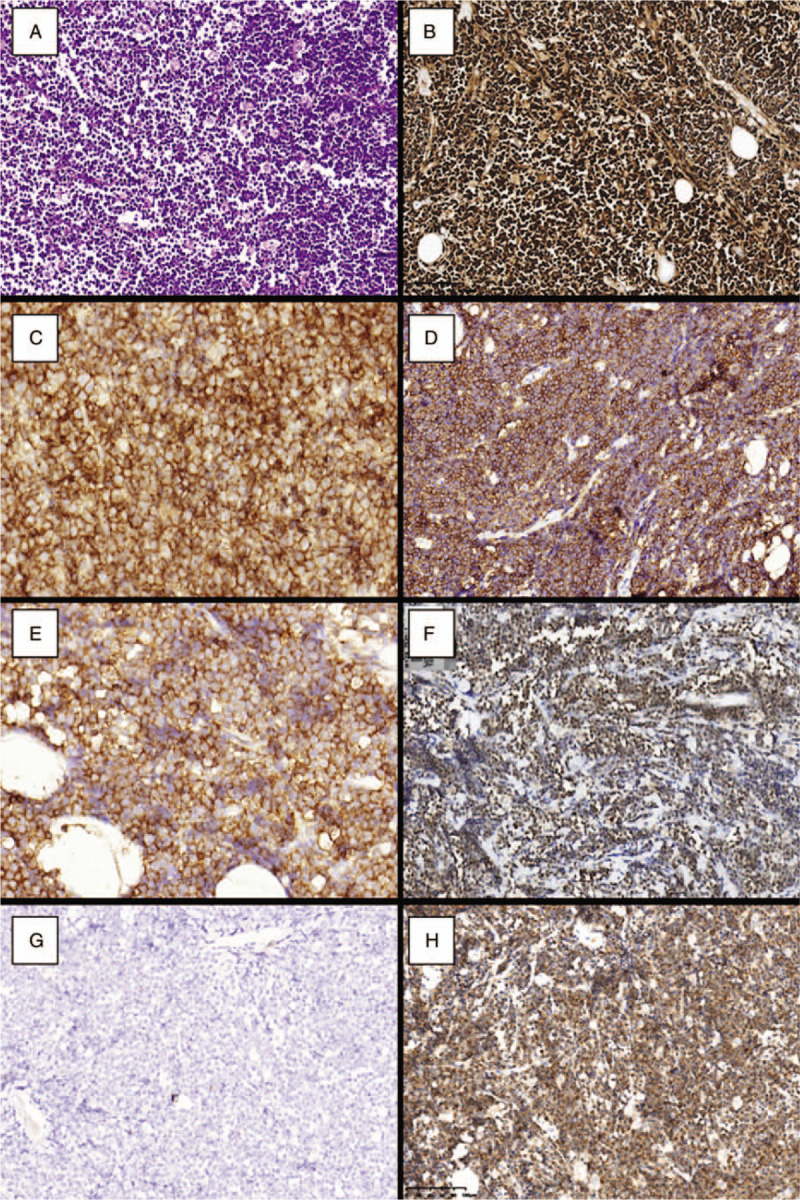
Medium-sized atypical lymphoid cells clusters and widespread abnormal lymphoid cells. Abnormal lymphocytes were interspersed with some tissue cells containing lightly stained cytoplasm. Numerous mitotic figures were also presented (A, × 200). The cells uniformly expressed CD19(C,×200), CD20(D,×200), confirming B-cell-derived tumor; expressed CD10(E,×200), MUM-1(F,×200), without expression of Bcl-6(G,×200), suggesting GCB. The proliferation rate was very high (ki-67>90%) (B,×200). IHC demonstrated an expression of Bcl-2 (H,×200) confirming the diagnosis of diffuse large B-cell lymphoma of cells (×200). IHC = immunohistochemistry, GCB = germinal-center type B-cell classification.

The 25-year-old married woman was sent to our hospital with a 7-week history of painless mass in the left breast. Seven weeks prior to this admission, she had been sent to a local hospital and treated with anti-inflammatory drugs for a presumed infection, but without any effects. Four years ago she was admitted to the hospital for rash on her face, elbow, and hands, laboratory findings showed a decrease in 3 lines, 24 hours urine protein significantly elevated, rheumatological profile revealed that anti-ANA, anti-dsDNA, antistreptolysin O were positive, liver and kidney function tests were normal, SLE and lupus nephritis were diagnosed. After a definite diagnosis, she was treated with mycophenolate mofetil (0.75 g/d), prednisone (1 mg/kg/d), hydroxychloroquine sulfate (0.4 g/d), and leflunomide (20 mg/d) to control the activity of SLE. After alleviating, the dose was reduced, with mycophenolate mofetil (0.5 g, bid), prednisone (15 mg/d), hydroxychloroquine sulfate (0.4 g/d) for maintenance treatment. After this admission, there was one mobile nodule in the left breast, and its surface had multiple necrotic and hemorrhagic foci on physical examination (Fig. 1). Ultrasonography of the breast revealed low echo nodule in the left breast with a volume of 4.2 × 1.8 cm approximately (Fig. 1). The left axillary lymph node was not palpable, while the right axillary and supraclavicular lymph nodes were not palpable. Molybdenum palladium roentgenograph of breast revealed an irregular soft tissue mass with a size of 4.3 × 3.7 cm in the upper quadrant of the left breast, slightly higher than the density of the gland (Fig. 1). Blood pressure was 160/110 mm Hg, and pulse was 109/min, temperature 36.6°C. Urinary tract ultrasonography showed bilateral kidney enlargement with diffuse lesions and mild hydronephrosis. The right kidney lower pole capsule was unclear. Laboratory findings showed as follows: protein excretion 1.03 g/24 h, white blood cells in urine (high power microscope) 10.42 (0–5), the serum myoglobulin 120.1 (<106) ng/mL, lactate dehydrogenase level 1493 (106–211) IU/L, creatinine 189.339 (46–92) μmol/L, the uric acid 687 (149–369) μmol/L, the urea 14.13 (2.5–6.1) mmol/L, and bicarbonate 16.7 (22–30) mmol/L. Unfortunately, with the disease progression, the content of creatinine, uric acid and urea in serum increased significantly (812.55 μmol/L, 1765 μmol/L, and 37.89 mmol/L, respectively), and the content of bicarbonate (10.2 mmol/L) decreased, which indicated that the patient had developed into renal insufficiency or even renal failure. The patient's family members were told about the disease condition. According to the above results, continued operation may aggravate the lupus activity and kidney damage, and should be delayed. However, the patient and her family members insisted on the mastectomy. Histologic examination revealed that medium-sized clusters and widespread abnormal lymphoid cells. Abnormal lymphocytes were interspersed with macrophages containing lightly stained cytoplasm. Numerous mitotic results were also presented in Figure 2. The immunohistochemistry (IHC) results showed the cells regularly expressed CD19, CD20, confirming B-cell-derived tumor; expressed CD10 and MUM-1, without Bcl-6, suggesting germinal-center type b-cell classification (Hans classification). The proliferation rate was very high (ki-67>90%). IHC demonstrated that the expression of Bcl-2 confirmed the diagnosis of diffuse large B-cell lymphoma of Cells (Fig. 2). Epstein–Barr virus (EBV)-encoded RNA by in-situ hybridization technology revealed that EBV was negative. Bone marrow puncture results showed that 36% of cancer cells were scattered on the edge or tail of the smear, suggesting that bone marrow metastasis occurred in the patient. Based on the morphology and immunohistochemistry, the diagnosis of primary breast diffuse large B-cell lymphoma was confirmed finally.

Results of her rheumatological profile revealed that the anti-ANA, anti-SS-A, and anti-Ro-52 were positive, while the anti-dsDNA, anti-SS-B, and anti-Sm antibody were all negative. The test for the anti-cardiolipin IgG/M was negative. C-reactive protein level 50.7 (normal: 0–1), C3 level 0.85 (normal: 0.9–1.8) g/L, C4 level 0.3 (normal: 0.1–0.4) g/dL, erythrocyte sedimentation rate 74 mm/h, immunoglobulin G (IgG) level 4.98 (normal: 8–16) g/L, and the blood platelet count 272 × 10^9^/L. According to the systemic lupus erythematosus disease activity score (SLEDAI), there might be a mild activity in lupus, but the renal function is damaged seriously, so we speculated that the deterioration of renal function may be mainly caused by infiltration of lymphoma. Unfortunately, the patient refused to undergo a kidney biopsy. With the development of the disease, the level of uric acid in the blood is 1765 μmol/L, and potassium 7.19 (3.5–5.3) mmol/L, phosphorus 5.95 (0.78–1.65) mmol/L, and calcium 1.59 (2.08–2.65) mmol/L. From these data indicate that the patient had a significant metabolic disorder, that is, tumor lysis syndrome that was characterized by acute renal failure and hyperkalemia. Treatment strategy with vincristine and dexamethasone was used to improve symptoms. However, the patient's renal function deteriorated and the blood potassium rose continuously, indicating that the treatment efficacy is not unsatisfactory. Therefore, she should be treated with hemodialysis and further treatment, but she and their family members refused the follow-up treatments. Unfortunately, the patient died 8 months after she was discharged from the hospital.

## 3 Discussion

Primary breast lymphoma (PBL) accounts for less than 1% of non-Hodgkin lymphoma (NHL), representing an unusual subgroup of NHL.^[9,10]^ The most common histology of PBL is DLBCL.^[11,12]^ Compared with the general DLBCL patients, PB-DLBCL cohort are more likely to relapse despite 2 years of event-free survival, by comparison, the recurrences of the DLBCL patients occur earlier, considering the above characteristics of PBL patients, so long-term follow-up review and reexamination are particularly important.^[13]^ Besides, PB-DLBCL has a high risk of contralateral breast failures with the recurrence rate of 21% in the group.^[13]^ Evidences had indicated that PB-DLBCL is an independent entity in clinic.^[14,15]^

SLE is a multisystemic relapsing and remitting autoimmune disease, involving various organs and tissues. SLE patients have a relatively higher incidence of leukemia and cancers of vulva, lung, thyroid, and liver. In addition, quadruple more likely for NHL were noted compared with healthy controls.^[2]^ DLBCL is the most frequent, accounting for nearly 90% in the cases.^[16]^

There are various possible explanations on the association between lymphoma and SLE, but some reasons are still controversial: First, the upregulation of cytokines. For instance, the cytokines-B cell activating factor /B lymphocyte stimulator (BAFF/BLyS) and proliferation inducing ligand (APRIL), which belong to the tumour necrosis factor (TNF) ligand superfamily, contribute to the survival and development of the B cell. B cell activating factor (BAFF) can stimulate the expression of pro-apoptotic molecules and decrease the expression of antiapoptotic molecules in mature B cells.^[17]^ Overexpression of APRIL was observed in SLE patients with DLBCL.^[18]^ Second, the disease activity. King and Costenbader ^[19]^ summarized 11 SLE patients who subsequently developed into NHL and showed this population had a moderate-intensity disease activity, while Bernatsky et al^[20]^ ascertained that there was no correlation between disease activity and lymphoma. In our case, the patient's renal function deteriorates continuously, and tumor lysis syndrome appeared in a short time after admission. However, according to the SLEDAI used to determine lupus activity in the rheumatology, there might be a mild activity in lupus, so we speculated that the continuous renal hypofunction may be mainly caused by infiltration of lymphoma. Unfortunately, however, the patient and her family refused to do a kidney biopsy. Third, immunosuppressive drugs. Multiple studies demonstrated SLE patients with a history of immunosuppressive drugs which contained mycophenolate mofetil cyclophosphamide, azathioprine, methotrexate, and glucocorticoids were inclined to develop into lymphoma.^[20,21]^ In our case, the patient was treated with prednisone, mycophenolate mofetil, hydroxychloroquine sulfate, and leflunomide to control the activity of lupus nephritis for 4 years, these drugs may promote the occurrence of lymphoma through immunosuppression and cytotoxicity.^[22]^ However, there were numerous researches that indicated that immunosuppressive medications did not promote the occurrence of lymphoma in SLE patients.^[19,23–25]^ Fourth, the functional defects of suppressor T-cell in the SLE patients. This deficiency of suppressor T-cell leads to the B lymphocytes excessive proliferation.^[26]^ Fifth, translocations involving the juxtaposition of an gene critical for immune cell function abutting a oncogene may promote the occurrence of lymphoma.^[27]^ Sixth, the continued infection of EBV may be a common cause for both the SLE and the B-cell lymphoma.^[2,28–30]^ Compared with healthy controls, patients with SLE had increased EBV at genetic and protein level even as well as viral load.^[31]^ Seventh, the chronic infection might be a crucial reason for lymphoma risk in autoimmune diseases like SLE.^[32]^

Based on the thorough analysis of existing research achievements, chemotherapy combined with radiation therapy (RT) is imperative for the treatment of PB-DLBCL. Notably, RT scope often includes the whole breast and the low axilla, as illustrated in recent guidelines for RT fields for extranodal lymphomas.^[11,33]^ The cohort of PB-DLBCL are prone to experience high rates of CNS relapse,^[11]^ but the benefit of central nervous system prophylaxis is unclear. With the increasing incidence of PBL, a suitable treatment for this rare NHL subtype is urgently needed.^[34]^

Numerous observational studies have confirmed that SLE patients had an increased risk of overall malignant tumors. PB-DLBCL is a rare occurrence in SLE patients. Therefore, a careful examination is very important in SLE cohort, as activity of the disease and malignancy may mimic each other. Meanwhile, when symptoms cannot be explained or insensitive to treatment, the occurrence of malignant tumors must be highly considered.

## Author contributions

**Data curation:** Feifei Shen, Shupeng Zhao, Fengjie Qi

**Methodology:** Gang Li, Huifeng Jiang, Fengjie Qi

**Writing – original draft:** Feifei Shen, Gang Li, MD, Huifeng Jiang

**Writing – review & editing:** Feifei Shen, Fengjie Qi
